# A Novel Active Fault-Tolerant Tracking Control for Robot Manipulators with Finite-Time Stability

**DOI:** 10.3390/s21238101

**Published:** 2021-12-03

**Authors:** Thanh Nguyen Truong, Anh Tuan Vo, Hee-Jun Kang, Mien Van

**Affiliations:** 1 Department of Electrical, Electronic and Computer Engineering, University of Ulsan, Ulsan 44610, Korea; thanhnguyen151095@gmail.com (T.N.T.); voanhtuan2204@gmail.com (A.T.V.); 2 School of Electronics, Electrical Engineering and Computer Science, Queen’s University Belfast, Belfast BT7 1NN, UK; m.van@qub.ac.uk

**Keywords:** fault tolerant control, fault detection observer, terminal sliding mode control, finite-time control theory, robot manipulators

## Abstract

Many terminal sliding mode controllers (TSMCs) have been suggested to obtain exact tracking control of robotic manipulators in finite time. The ordinary method is based on TSMCs that secure trajectory tracking under the assumptions such as the known robot dynamic model and the determined upper boundary of uncertain components. Despite tracking errors that tend to zero in finite time, the weakness of TSMCs is chattering, slow convergence speed, and the need for the exact robot dynamic model. Few studies are handling the weakness of TSMCs by using the combination between TSMCs and finite-time observers. In this paper, we present a novel finite-time fault tolerance control (FTC) method for robotic manipulators. A finite-time fault detection observer (FTFDO) is proposed to estimate all uncertainties, external disturbances, and faults accurately and on time. From the estimated information of FTFDO, a novel finite-time FTC method is developed based on a new finite-time terminal sliding surface and a new finite-time reaching control law. Thanks to this approach, the proposed FTC method provides a fast convergence speed for both observation error and control error in finite time. The operation of the robot system is guaranteed with expected performance even in case of faults, including high tracking accuracy, small chattering behavior in control input signals, and fast transient response with the variation of disturbances, uncertainties, or faults. The stability and finite-time convergence of the proposed control system are verified that they are strictly guaranteed by Lyapunov theory and finite-time control theory. The simulation performance for a FARA robotic manipulator proves the proposed control theory’s correctness and effectiveness.

## 1. Introduction

Robot manipulators are widely used in the industrial manufacturing and service industries due to their persistence in operation, repetitive works, flexibility, heavy jobs, as well as requirements of high accuracy. Safety and high tracking performance are expected for many tasks that are a challenge in robot operation. The main challenges that interfere with the safety and operation of the robot can be included complex system dynamics, nonlinearities, frictions, external disturbances, uncertainties, faults, etc. For conventional control methods, they can provide the expected performance and safety under the influence of uncertainties when no faults occur in the system. Once faults occur, safety is seriously affected and control performance is reduced, leading to system instability. These affect the quality of the product, increasing the danger in the work environment with the presence of people. Recently, fault diagnosis (FD) and fault-tolerant control (FTC) have attracted a lot of attention for detecting faults and maintaining the expected performance of Markovian jump systems [1], nonlinear systems [2], or robot manipulators in the existence of multiple faults.

In recent years, the FD/FTC methods of robot manipulators have been extensively investigated [3,4,5,6,7]. Those methods have also achieved remarkable results. Unfortunately, the existing FD/FTC methods have drawbacks that need to be handled. The FTC systems can develop from two following types, including active FTC (AFTC) [6,8] and passive FTC (PFTC) [9,10].

For PFTC, it provides a fast response to the effects of disturbances/faults. This method doesn’t demand feedback information of disturbances/faults from FD. However, it needs to be known their upper bound value. In comparison with PFTC, AFTC provides outstanding performance when feedback information of faults from the FD is provided correctly. Therefore, some observers have been proposed for fault diagnosis to obtain higher exact information of the faults, such as high-gain observers [11,12], neural network observers [13,14], sliding mode observers (SMOs) [15,16], higher-order sliding mode observers (HOSMOs) [17,18,19], fuzzy logic observers [20,21], disturbance observers [22,23,24,25], etc. To provide accurate and timely fault information, the convergence of observers needs to be ensured within finite time. Among them, SMOs and HOSMOs obtain better performance than the remaining observers. However, SMOs generate chattering. HOSMO is capable to provide finite-time convergence and chattering reduction can also be achieved. Therefore, the advantages of HOSMO have been inherited in this paper to construct a finite-time fault diagnosis observer. Thus, external disturbances or faults can be identified by an observer. The next goal is to reconstruct a suitable controller from the information of the faults or external disturbances to resolve the instability caused by the influences of faults or disturbances while maintaining the desired performance for the system.

We always know that the robot is a nonlinear system; it is difficult for the fundamental methods such as inverse dynamic control [26], PID [27], or the computed torque control (CTC) [28], to achieve high accuracy and low sensitivity to external disturbances. In this case, the nonlinear controllers seem to be more suitable than the linear ones. The prominent nonlinear controller widely used in practice is sliding mode control (SMC) [29,30].

Nowadays, SMC has had its application extended to FTC systems [31,32]. SMC is designed to exhibit robust control performance under bounded disturbances, uncertainties, or faults. Unfortunately, SMC only gradually reaches the equilibrium point when the convergence time tends to infinity. As with SMO, SMC also generates a lot of unexpected chattering [33].

To increase the convergent rate of SMC, terminal sliding mode (TSM) controllers were introduced [34,35,36] and extended to FTC systems [37,38,39]. TSM control obtains finite-time stability caused by its nonlinear sliding surface, which provides lower control errors and a higher convergence rate than those of conventional linear sliding mode (LSM) control. The advantages of TSM control have been confirmed in many applications of FTC systems [6,19,38,40,41]. Nevertheless, excessively fast convergence is associated with more serious chattering, which is dangerous and corrupting for a real application. Furthermore, the weakness of TSMC is that the convergence time will be greatly increased when the initial values of the system states are far from the origin. This is also one of the motivations for us to design a new sliding surface to overcome the slow convergence in the above case.

Many studies have been performed to explore approaches in reducing the chattering that happens in SMC and TSMC. Usage of the saturation function instead of the sign function can reduce chattering significantly. However, the tracking accuracy would also be reduced in this case [42]. A few intelligent controllers have been adopted to effectively solve chattering problems [43,44,45]. The application of intelligent methods into controlling is also not easy, since they often need a lot of parameters or complex tuning methods for the parameters. Therefore, it increases the computation burden. The application of a disturbance/perturbation observer as HOSMO is also effective in reducing chattering and increasing tracking accuracy, as discussed above.

From the above discussion, it is really necessary to develop the FTC system for the robot. The FTC system is a combination of a finite time observer and advanced TSMC. The system can improve the slow convergence of the conventional TSMC when the initial values of the system states are far from the origin. The proposed control system guarantees a fast convergence rate for both observation error and control error in finite time. It exhibits high control precision and robustness against disturbance, uncertainties and maintains the desired performance for the system in case of faults. In addition, the occurrence of chattering in the control input has been significantly minimized. In summary, the stability of the new FTC system is strictly guaranteed in finite time, and it is reinforced by the Lyapunov stability theory. The simulation and comparison performance among some state-of-art methods for a FARA robotic manipulator proves the proposed control theory’s correctness and effectiveness.

The rest of this article is organized as follows. Section 2 of this article presents problem statements and notations. In Section 3, the proposed control design procedure and stability analysis are described. Simulation results that demonstrate the effectiveness of the new proposed controller are discussed in Section 4. Finally, the remarkable conclusions of this study are presented in Section 5.

## 2. Notations and Problem Statements

### 2.1. Notations

A symbol list is provided in Table 1 to convenience the reader as follows

The functions which are utilized in the subsequent content are defined as follows:

(1) $$sign\left(q_{i}\right)=\left\{\begin{array}{c} 1\;\mathrm{if}\;q_{i}>0\\ 0\;\mathrm{if}\;q_{i}=0\\ -1\;\mathrm{if}\;q_{i}<0 \end{array}\right.\;$$

(2) $$sign\left(q\right)={\left[\begin{array}{ccc} sign\left(q_{1}\right), & \ldots & ,\;sign\left(q_{n}\right) \end{array}\right]}^{T}$$

(3) $$sig{\left(q_{i}\right)}^{\sigma_{i}}={\left|q_{i}\right|}^{\sigma_{i}}sign\left(q_{i}\right)\;$$

(4) $$\frac{d}{dt}\left(sig{\left(q_{i}\right)}^{\sigma_{i}}\right)=\sigma_{i}{\left|q_{i}\right|}^{\sigma_{i}-1}{\dot{q}}_{i}$$

(5) $$sig{\left(q\right)}^{\sigma }={\left[\begin{array}{ccc} {\left|q_{1}\right|}^{\sigma_{1}}sign\left(q_{1}\right), & \ldots & ,\;{\left|q_{n}\right|}^{\sigma_{n}}sign\left(q_{n}\right) \end{array}\right]}^{T}$$

(6) $$diag\left(q\right)=\left[\begin{array}{cccc} q_{1} & 0 & 0 & 0\\ 0 & q_{2} & 0 & 0\\ 0 & 0 & \ddots & 0\\ 0 & 0 & 0 & q_{n} \end{array}\right]$$

(7) $$⎢⎢q⎥⎥=\sqrt{\overset{n}{\underset{i=1}{\sum }}\left|q_{i}\right|}$$

### 2.2. Problem Statements

The dynamic model of an n-degree-of-freedom robotic manipulator is written as follows:

(8) $$M\left(\theta \right)\ddot{\theta }+C\left(\theta ,\dot{\theta }\right)\dot{\theta }+G\left(\theta \right)+F\left(\dot{\theta }\right)+T_{d}\left(t\right)=\tau -\sigma \left(t-T_{F}\right)\Gamma \left(\theta ,\dot{\theta },\tau \right)$$

where $\theta ,\dot{\theta },\ddot{\theta }\in {\mathbb{R}}^{n}$ are angle position, angular velocity, and angular acceleration of the joints, respectively. $M\left(\theta \right)=\hat{M}\left(\theta \right)+\Delta M\left(\theta \right)\in {\mathbb{R}}^{n\times n}$ is the actual inertia matrix, $C\left(\theta ,\dot{\theta }\right)=\hat{C}\left(\theta ,\dot{\theta }\right)+\Delta C\left(\theta ,\dot{\theta }\right)\in {\mathbb{R}}^{n\times n}$ is the actual centrifugal and Coriolis force matrix, $G\left(\theta \right)=\hat{G}\left(\theta \right)+\Delta G\left(\theta \right)\in {\mathbb{R}}^{n}$ is the vector of actual gravity, $F\left(\dot{\theta }\right)\in {\mathbb{R}}^{n}$ is the vector of friction force, $T_{d}\left(t\right)\in {\mathbb{R}}^{n}$ is the vector of external disturbance, $\tau \in {\mathbb{R}}^{n}$ is the vector of control input torque. $\hat{M}\left(\theta \right),\;\hat{C}\left(\theta ,\dot{\theta }\right)$, and $\hat{G}\left(\theta \right)$ are the estimated matrices of $M\left(\theta \right),\;C\left(\theta ,\dot{\theta }\right),$ and $G\left(\theta \right),$ respectively. $\Delta M\left(\theta \right),\;\Delta C\left(\theta ,\dot{\theta }\right)$, and $\Delta G\left(\theta \right)$ are the estimation error matrices of the dynamic model. $\Gamma \left(\theta ,\dot{\theta },\tau \right)\in {\mathbb{R}}^{n}$ represents the unexpected fault influencing the robotic manipulator. $\sigma \left(t-T_{F}\right)\in {\mathbb{R}}^{n\times n}$ is the time profile of the faults, and $T_{F}$ is the time when the faults occur.

The time profile of fault is configured as a diagonal matrix as the following form:

(9) $$\sigma \left(t-T_{F}\right)=diag\left\{\sigma_{1}\left(t-T_{F1}\right),\ldots ,\sigma_{n}\left(t-T_{Fn}\right)\right\}$$

where $\sigma_{i}$ represents the effect of fault on $i^{th}$ state equation and it is defined by

(10) $$\sigma_{i}\left(t-T_{Fi}\right)=\left\{\begin{array}{cc} 0 & \mathrm{if}\;t<T_{Fi}\\ 1-\exp\left(-\ell_{i}\left(t-T_{F_{i}}\right)\right) & \mathrm{if}\;t>T_{Fi} \end{array}\right.$$

in which $\ell_{i}$ is the developing rate coefficient of the $i^{th}$ fault.

Let $x_{1}=\theta$ and $x_{2}=\dot{\theta }$, the dynamic model in Equation (8) is transformed into the second-order state-space model as follows:

(11) $$\left\{\begin{array}{l} {\dot{x}}_{1}=x_{2}\\ {\dot{x}}_{2}=P\left(x\right)+B\left(x\right)u+D \end{array}\right.$$

where $x={\left[\begin{array}{cc} x_{1}^{T} & x_{2}^{T} \end{array}\right]}^{T}$ is a system state vector, $P\left(x\right)=\hat{M}{\left(\theta \right)}^{-1}\left(-\hat{C}\left(\theta ,\dot{\theta }\right)\dot{\theta }-\hat{G}\left(\theta \right)\right)$, $B\left(x\right)=\hat{M}{\left(\theta \right)}^{-1}$, u=τ is a vector of control input torque, $D=\hat{M}{\left(\theta \right)}^{-1}\left(-\Delta M\left(\theta \right)\ddot{\theta }-\Delta C\left(\theta ,\dot{\theta }\right)\dot{\theta }-\Delta G\left(\theta \right)-F\left(\dot{\theta }\right)-T_{d}\left(t\right)-\sigma \left(t-T_{F}\right)\Gamma \left(\theta ,\dot{\theta },\tau \right)\right)$ represents the whole of uncertainties, external disturbance, and fault which is constructed by robot’s dynamic model uncertainties ($\Delta M\left(\theta \right),\;\Delta C\left(\theta ,\dot{\theta }\right),\;\Delta G\left(\theta \right)$), friction force ($F\left(\dot{\theta }\right)$), external disturbance ($T_{d}\left(t\right)$), and fault ($\Gamma \left(\theta ,\dot{\theta },\tau \right)$).

**Assumption** **1:** *The whole uncertainty components and its time derivative are bounded by*:

(12) $$\left\{\begin{array}{c} ⎢⎢D⎥⎥\leq \Omega_{1}\\ \dot{⎢⎢D⎥⎥}\leq \Omega_{2} \end{array}\right.$$

*where*$\Omega_{1}$*and*$\Omega_{2}$*are positive constants*.

From Equation (11), we can see that the dynamic model of the system contains uncertainty, external disturbance, and fault. Thus, achieving stability in a finite time while maintaining the desired performance under fault conditions remains a major challenge. Therefore, this paper designs a fault-tolerant controller to overcome the mentioned challenge.

## 3. The Proposed Control Design Procedure

In this section, we present a new FTC method, which provides a finite convergence time, high trajectory accuracy, significantly reduces chattering behavior in the control input signals, and achieves strong stability for the robotic manipulator system.

First, a fault diagnosis observer (FDO) based on HOSMO [17] is developed, which can approximate the uncertainties as well as the external noise, and faults within finite time. Therefore, it can provide accurate information into the control system in a timely and accurate manner. Second, a novel fast terminal sliding mode (FTSM) control is proposed based on a novel FTSM surface which enhances the advantages of FTSM surface, and a novel fast-reaching control law (FRCL). Finally, the finite-time stability of the whole control system is proved by Lyapunov criteria.

### 3.1. Design the FDO

Based on the robotic system in Equation (11), an FDO is designed to estimate the whole uncertainties and faults as follows:

(13) $$\left\{\begin{array}{c} {\tilde{x}}_{1}=x_{1}-{\hat{x}}_{1}\\ {\dot{\hat{x}}}_{1}={\hat{x}}_{2}+\varphi sig{\left({\tilde{x}}_{1}\right)}^{\frac{2}{3}}\\ {\dot{\hat{x}}}_{2}=\hat{D}+P\left(x\right)+B\left(x\right)u+\psi sig{\left({\tilde{x}}_{1}\right)}^{\frac{1}{3}}\\ \dot{\hat{D}}=\varrho sign\left({\tilde{x}}_{1}\right) \end{array}\right.$$

where ${\hat{x}}_{1}$ is estimated value of the position $x_{1}$**,** ${\hat{x}}_{2}$ is estimated value of velocity $x_{2}$, $\hat{D}$ is estimated value of the whole uncertainties and faults D, $\varphi =diag\left\{\varphi_{1},\;\ldots ,\;\varphi_{n}\right\}$, $\varphi_{i}>0,\;\psi =diag\left\{\psi_{1},\;\ldots ,\;\psi_{n}\right\}$**,** $\psi_{i}>0$, $\varrho =diag\left\{\varrho_{1},\;\ldots ,\;\varrho_{n}\right\},\;\varrho_{i}>0$.

By subtracting Equation (13) from Equation (11), we can obtain the estimation errors as follows:

(14) $$\left\{\begin{array}{c} {\dot{\tilde{x}}}_{1}=-\varphi sig{\left({\tilde{x}}_{1}\right)}^{\frac{2}{3}}+{\tilde{x}}_{2}\\ {\dot{\tilde{x}}}_{2}=-\psi sig{\left({\tilde{x}}_{1}\right)}^{\frac{1}{3}}+\tilde{D}\\ \dot{\tilde{D}}=-\varrho sign\left({\tilde{x}}_{1}\right)+\dot{D} \end{array}\right.$$

where ${\tilde{x}}_{2}=x_{2}-{\hat{x}}_{2}$, $\tilde{D}=D-\hat{D}$.

**Assumption** **2:** *Assume that the estimation error of the whole uncertainty and fault is bounded by*:

(15) $$\tilde{⎢⎢D⎢⎢}\leq J,$$

*where*J*is a positive constant*.

Equation (14) is finite-time stable, which has been already proved in [46,47]. Therefore, by choosing appropriate gains φ, ψ, and ϱ (the readers can refer to the existing study [48]), we can determine that ${\tilde{x}}_{1}$, ${\tilde{x}}_{2}$, and $\tilde{D}$ will converge to zero in finite time $t>T_{o}=\underset{1\leq i\leq n}{max}\left\{T_{2}{}_{i}\;\right\}$. It can be observed that after a finite time $T_{o}$, ${\hat{x}}_{1}=x_{1}$, ${\hat{x}}_{2}=x_{2}$ and $\hat{D}=D$.

### 3.2. Design of Novel Finite-Time FTSM Surface

We define the tracking position error, and the tracking velocity error as follows:

(16) $$\begin{array}{c} x_{e_{i}}=x_{1_{i}}-\theta_{d_{i}}\\ x_{de_{i}}=x_{2_{i}}-{\dot{\theta }}_{d_{i}},\;i=1,\ldots ,\;n \end{array}$$

where $\theta_{d}={\left[\theta_{d_{1}},\;\ldots ,\;\theta_{d_{n}}\right]}^{T}\in {\mathbb{R}}^{n},{\dot{\theta }}_{d}={\left[{\dot{\theta }}_{d_{1}},\;\ldots ,\;{\dot{\theta }}_{d_{n}}\right]}^{T}\in {\mathbb{R}}^{n}$ with $\theta_{d_{i}}$ and ${\dot{\theta }}_{d_{i}}$ respectively are desired position and desired velocity at $i^{th}$ joint. And $x_{1}={\left[x_{1_{1}},\;\ldots ,\;x_{1_{n}}\right]}^{T}\in {\mathbb{R}}^{n}$, $x_{2}={\left[x_{2_{1}},\;\ldots ,\;x_{2_{n}}\right]}^{T}\in {\mathbb{R}}^{n}$ with $x_{1_{i}}$ and $x_{2_{i}}$ respectively are actual position and actual velocity at $i^{th}$ joint.

The FTSMC concept first introduced in [49] can be presented as follows:

(17) $$s_{i}=x_{de_{i}}+\lambda_{i}sig{\left(x_{e_{i}}\right)}^{\alpha_{i}}+\omega_{i}sig{\left(x_{e_{i}}\right)}^{\beta_{i}},\;i=1,\ldots ,\;n$$

where $\lambda_{i}>0,\;\omega_{i}>0,$ $\alpha_{i}>1$, $0<\beta_{i}<1$.

From Equation (17), when $s_{i}=0\;\left(i=1,\ldots ,\;n\right)$, the sliding motion formula is given by:

(18) $$x_{dei}=-\lambda_{i}sig{\left(x_{ei}\right)}^{\alpha_{i}}-\omega_{i}sig{\left(x_{ei}\right)}^{\beta_{i}},\;i=1,\ldots ,\;n$$

Equation (18) shows that the first term plays an important role when $x_{ei}$ is far away from 0. The second term plays an important role when $x_{ei}$ is near 0.

To further enhance the performance of the FTSMC, an innovated sliding mode surface is proposed as follows:

(19) $$s_{i}=x_{de_{i}}+\lambda_{i}^{*}sig{\left(x_{e_{i}}\right)}^{\alpha_{i}}+\omega_{i}^{*}sig{\left(x_{e}{}_{i}\right)}^{\beta_{i}},\;i=1,\ldots ,n$$

where $\lambda_{i}^{*}=\frac{2\lambda_{i}}{1+\exp\left(-\eta_{i}\left(\left|x_{e_{i}}\right|-\gamma_{i}\right)\right)}$, $\omega_{i}^{*}=\frac{2\omega_{i}}{1+\exp\left(\sigma_{i}\left(\left|x_{e_{i}}\right|-\gamma_{i}\right)\right)}$, $\lambda_{i}>0,\eta_{i}>0,\omega_{i}>0,\sigma_{i}>0$, $\alpha_{i}>1$, $0<\beta_{i}<1$, $\gamma_{i}={\left(\omega_{i}/\lambda_{i}\right)}^{1/\left(\alpha_{i}-\beta_{i}\right)}$.

When $s_{i}=0\;\left(i=1,\ldots ,\;n\right)$, then the sliding motion equation is formulated as:

(20) $$x_{de_{i}}=-\lambda_{i}^{*}sig{\left(x_{e_{i}}\right)}^{\alpha_{i}}-\omega_{i}^{*}sig{\left(x_{e_{i}}\right)}^{\beta_{i}},\;i=1,\ldots ,\;n$$

Equation (20) differs from Equation (18) in that the coefficients of the sliding surface ($\lambda_{i}^{*},\;\omega_{i}^{*}$) are dynamically changed with $\left|x_{e_{i}}\right|$.

**Remark** **1:** *When*$\left|x_{e_{i}}\right|\geq \gamma_{i}$*the first term of Equation (20) plays the main role, and the remaining term of Equation (20) plays the secondary role. On contrary, when*$\left|x_{e_{i}}\right|<\gamma_{i}$*the first term of Equation (20) plays a smaller role, and the second term of Equation (20) plays the main role. Overall, this approach aims to strengthen the effect of the term that has a major role, while weakening the effect of the term that has a minor role, which will improve the transient response performance of the control system*.

**Lemma** **1.** *Consider a scalar differential equation as follows*:

(21) $$x_{de_{i}}=-\lambda_{i}^{*}sig{\left(x_{e_{i}}\right)}^{\alpha_{i}}-\omega_{i}^{*}sig{\left(x_{e_{i}}\right)}^{\beta_{i}}$$

*where*$\lambda_{i}^{*}=\frac{2\lambda_{i}}{1+exp\left(-\eta_{i}\left(\left|x_{e_{i}}\right|-\gamma_{i}\right)\right)}$, $\omega_{i}^{*}=\frac{2\omega_{i}}{1+exp\left(\sigma_{i}\left(\left|x_{e_{i}}\right|-\gamma_{i}\right)\right)}$, $\lambda_{i}>0,\eta_{i}>0,\omega_{i}>0,\sigma_{i}>0$, $\alpha_{i}>1$, $0<\beta_{i}<1$, $\gamma_{i}={\left(\omega_{i}/\lambda_{i}\right)}^{1/\left(\alpha_{i}-\beta_{i}\right)}$. *Then, the system (21) is a globally finite time-stable, and the convergence time* $T_{s_{i}}$*is bounded by*

(22) $$T_{s_{i}}=\frac{1}{\lambda_{i}\left(-\alpha_{i}+1\right)}\left({\left|x_{e_{i}}\left(0\right)\right|}^{-\alpha_{i}+1}-\gamma_{i}^{-\alpha_{i}+1}\right)+\frac{1}{\omega_{i}\left(-\beta_{i}+1\right)}{\left|\gamma_{i}\right|}^{-\beta_{i}+1}$$

**Proof:**  Select the Lyapunov function $V_{2_{i}}=0.5x_{e_{i}}^{2}\;\left(i=1,\;\ldots ,\;n\right)$, then the time derivative of $V_{2_{i}}$ is

(23) $${\dot{V}}_{2_{i}}=x_{e_{i}}x_{de_{i}}=x_{e_{i}}\left(-\lambda_{i}^{*}sig{\left(x_{e_{i}}\right)}^{\alpha_{i}}-\omega_{i}^{*}sig{\left(x_{e_{i}}\right)}^{\beta_{i}}\right)\;=-\lambda_{i}^{*}{\left|x_{e_{i}}\right|}^{\alpha_{i}+1}-\omega_{i}^{*}{\left|x_{e_{i}}\right|}^{\beta_{i}+1}<0$$

It can be shown that $V_{2_{i}}>0$ and ${\dot{V}}_{2_{i}}<0$. Therefore, the state variables $x_{e_{i}}$ and $x_{de_{i}}$ can converge to the equilibrium point. When $\left|x_{e}{}_{i}\left(0\right)\right|>\gamma_{i}$, the sliding motion is divided into two stages: $x_{e}{}_{i}\left(0\right)\to \left|x_{e_{i}}\right|=\gamma_{i}$ and $\left|x_{e}{}_{i}\right|=\gamma_{i}\to 0$. Consequently, the setting time can be calculated as follows.

Stage 1: $x_{e_{i}}\left(0\right)\to \left|x_{e_{i}}\right|=\gamma_{i}$. The first term of Equation (21) plays the main role:

(24) $$\int_{0}^{t_{s1_{i}}}dt=\int_{x_{e_{i}}\left(0\right)}^{\gamma_{i}}\frac{1}{-\lambda_{i}^{*}{\left|x_{e_{i}}\right|}^{\alpha_{i}}-\omega_{i}^{*}{\left|x_{e}{}_{i}\right|}^{\beta_{i}}}d\left(\left|x_{e}{}_{i}\right|\right)\;<\int_{\gamma_{i}}^{x_{e_{i}}\left(0\right)}\frac{1}{\lambda_{i}{\left|x_{e_{i}}\right|}^{\alpha_{i}}}d\left(\left|x_{e}{}_{i}\right|\right)={\left[\frac{{\left|x_{e_{i}}\right|}^{-\alpha_{i}+1}}{\lambda_{i}\left(-\alpha_{i}+1\right)}\right]}_{\gamma_{i}}^{x_{e_{i}}\left(0\right)}=\frac{1}{\lambda_{i}\left(-\alpha_{i}+1\right)}\left({\left|x_{e_{i}}\left(0\right)\right|}^{-\alpha_{i}+1}-\gamma_{i}^{-\alpha_{i}+1}\right)$$

Stage 2: $\left|x_{e_{i}}\right|=\gamma_{i}\to x_{e_{i}}=0$. The second term of Equation (21) plays the main role:

(25) $$\int_{0}^{t_{s2_{i}}}dt=\int_{\gamma_{i}}^{0}\frac{1}{-\lambda_{i}^{*}{\left|x_{e_{i}}\right|}^{\alpha_{i}}-\omega_{i}^{*}{\left|x_{e}{}_{i}\right|}^{\beta_{i}}}d\left(\left|x_{e}{}_{i}\right|\right)\;<\int_{0}^{\gamma_{i}}\frac{1}{\omega_{i}{\left|x_{e}{}_{i}\right|}^{\beta }}d\left(\left|x_{e}{}_{i}\right|\right)={\left[\frac{{\left|x_{e}{}_{i}\right|}^{-\beta_{i}+1}}{\omega_{i}\left(-\beta_{i}+1\right)}\right]}_{0}^{\gamma_{i}}=\frac{1}{\omega_{i}\left(-\beta_{i}+1\right)}{\left|\gamma_{i}\right|}^{-\beta_{i}+1}$$

Thus, the sum of the convergence time is calculated as:

(26) $$T_{s_{i}}=t_{s1_{i}}+t_{s2_{i}}=\frac{1}{\lambda_{i}\left(-\alpha_{i}+1\right)}\left({\left|x_{e_{i}}\left(0\right)\right|}^{-\alpha_{i}+1}-\gamma_{i}^{-\alpha_{i}+1}\right)+\frac{1}{\omega_{i}\left(-\beta_{i}+1\right)}{\left|\gamma_{i}\right|}^{-\beta_{i}+1}$$

The proof is completed. □

From Lemma 1, the convergence time of the system (20) is calculated as:

(27) $$T_{s}=\underset{1\leq i\leq n}{max}\left\{T_{s_{i}}\;\right\}=\underset{1\leq i\leq n}{max}\left\{\frac{1}{\lambda_{i}\left(-\alpha_{i}+1\right)}\left({\left|x_{e_{i}}\left(0\right)\right|}^{-\alpha_{i}+1}-\gamma_{i}^{-\alpha_{i}+1}\right)+\frac{1}{\omega_{i}\left(-\beta_{i}+1\right)}{\left|\gamma_{i}\right|}^{-\beta_{i}+1}\;\right\}$$

### 3.3. Design of Proposed Controller

To obtain the control laws for FTC of robot manipulators, the control design procedure is performed below.

Computing the time derivative of the proposed sliding surface in Equation (19) yields

(28) $${\dot{s}}_{i}={\dot{x}}_{de_{i}}+\frac{2\lambda_{i}\alpha_{i}}{1+\exp\left(-\eta_{i}\left(\left|x_{e_{i}}\right|-\gamma_{i}\right)\right)}{\left|x_{e_{i}}\right|}^{\alpha_{i}-1}x_{de_{i}}+\frac{2\lambda_{i}\eta_{i}\exp\left(-\eta_{i}\left(\left|x_{e_{i}}\right|-\gamma_{i}\right)\right)}{{\left(1+\exp\left(-\eta_{i}\left(\left|x_{e_{i}}\right|-\gamma_{i}\right)\right)\right)}^{2}}{\left|x_{e_{i}}\right|}^{\alpha_{i}}x_{de_{i}}\;+\frac{2\omega_{i}\beta_{i}}{1+\exp\left(\sigma_{i}\left(\left|x_{e_{i}}\right|-\gamma_{i}\right)\right)}{\left|x_{e_{i}}\right|}^{\beta_{i}-1}x_{de_{i}}-\frac{2\omega_{i}\sigma_{i}\exp\left(\sigma_{i}\left(\left|x_{e_{i}}\right|-\gamma_{i}\right)\right)}{{\left(1+\exp\left(\sigma_{i}\left(\left|x_{e_{i}}\right|-\gamma_{i}\right)\right)\right)}^{2}}{\left|x_{e_{i}}\right|}^{\beta_{i}}x_{de_{i}},\;i=1,\ldots ,\;n$$

The vector form of Equation (28) is rewritten as:

(29) $$\dot{s}={\dot{x}}_{de}+M={\dot{x}}_{2}-{\ddot{x}}_{d}+M$$

where $M={\left[\begin{array}{ccc} M_{1} & \ldots & M_{n} \end{array}\right]}^{T}\in {\mathbb{R}}^{n},\;M_{i}=\frac{2\lambda_{i}\alpha_{i}}{1+\exp\left(-\eta_{i}\left(\left|x_{e_{i}}\right|-\gamma_{i}\right)\right)}{\left|x_{e_{i}}\right|}^{\alpha_{i}-1}x_{de_{i}}+\frac{2\lambda_{i}\eta_{i}\exp\left(-\eta_{i}\left(\left|x_{e_{i}}\right|-\gamma_{i}\right)\right)}{{\left(1+\exp\left(-\eta_{i}\left(\left|x_{e_{i}}\right|-\gamma_{i}\right)\right)\right)}^{2}}{\left|x_{e_{i}}\right|}^{\alpha_{i}}x_{de_{i}}+\frac{2\omega_{i}\beta_{i}}{1+\exp\left(\sigma_{i}\left(\left|x_{e_{i}}\right|-\gamma_{i}\right)\right)}{\left|x_{e_{i}}\right|}^{\beta_{i}-1}x_{de_{i}}-\frac{2\omega_{i}\sigma_{i}\exp\left(\sigma_{i}\left(\left|x_{e_{i}}\right|-\gamma_{i}\right)\right)}{{\left(1+\exp\left(\sigma_{i}\left(\left|x_{e_{i}}\right|-\gamma_{i}\right)\right)\right)}^{2}}{\left|x_{e_{i}}\right|}^{\beta_{i}}x_{de_{i}},\;i=1,\ldots ,\;n$.

Substituting Equation (11) into Equation (29), we obtain

(30) $$\dot{s}=P\left(x\right)+B\left(x\right)u+D-{\ddot{x}}_{d}+M$$

To obtain a faster convergence for the system trajectory state to the designed sliding surface, a novel finite-time FRCL is proposed as

(31) $$\dot{s}=-Rsig{\left(s\right)}^{m}-Ksig{\left(s\right)}^{n}-Jsign\left(s\right)$$

where $R=diag\left\{\frac{2R_{1}}{1+\exp\left(-p_{1}\left(\left|s_{1}\right|-\zeta_{1}\right)\right)},\;\ldots ,\frac{2R_{n}}{1+\exp\left(-p_{n}\left(\left|s_{n}\right|-\zeta_{n}\right)\right)}\;\right\}\in {\mathbb{R}}^{n\times n}$, $R_{i}>0,\;p_{i}>0,\;K=diag\left\{\frac{2K_{1}}{1+\exp\left(q_{1}\left(\left|s_{1}\right|-\zeta_{1}\right)\right)},\;\ldots ,\frac{2K_{n}}{1+\exp\left(q_{n}\left(\left|s_{n}\right|-\zeta_{n}\right)\right)}\;\right\}\in {\mathbb{R}}^{n\times n}$, $K_{i}>0$, $q_{i}>0,$ m and n are parameter vectors with the element as $m_{i}>1$, $0<n_{i}<1$, $\zeta_{i}={\left(K_{i}/R_{i}\right)}^{1/\left(m_{i}-n_{i}\right)}$.

It is seen that when $\left|s_{i}\right|\geq \zeta_{i}$ or $\left|s_{i}\right|<\zeta_{i}$ the role of each term in Equation (31) plays the same role as stated in Remark 1. Thus, the system could quickly reach the surface in finite time.

Based on the output of the observer in Equation (13), substituting Equation (30) into Equation (31), a novel FTC law is designed as follows:

(32) $$\left\{\begin{array}{c} u=B^{-1}\left(x\right)\left(u_{eq}+u_{r}-\hat{D}\right)\\ u_{eq}={\ddot{x}}_{d}-P\left(x\right)-M\\ u_{r}=-Rsig{\left(s\right)}^{m}-Ksig{\left(s\right)}^{n}-Jsign\left(s\right) \end{array}\right.$$

**Figure 1:**
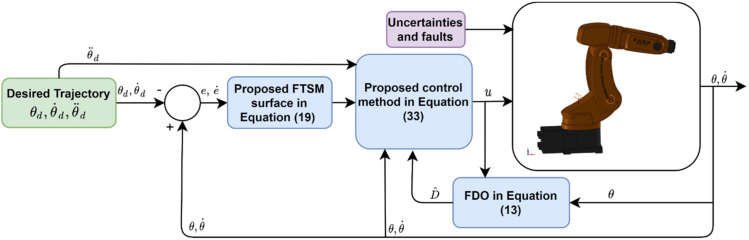
The block diagram of the proposed control method.

The block diagram of the proposed control method is illustrated in Figure 1.

The control design procedure can be summarized in the following theorem.

**Theorem** **1.** *Consider the robot system stated in Equation (11) if the proposed control laws are constructed as in Equation (32) which is formed from data of observer in Equation (13), the proposed sliding surface in Equation (19), and a novel finite-time reaching control law in Equation (31), then the system is globally finite-time stable*.

**Proof** **of** **Theorem** **1.** Substituting the control input in Equation (32) into Equation (30), we gain

(33) $$\dot{s}=D-\hat{D}-Rsig{\left(s\right)}^{m}-Ksig{\left(s\right)}^{n}-Jsign\left(s\right)$$

An element of the vector in Equation (33) is represented as

(34) $${\dot{s}}_{i}=D_{i}-{\hat{D}}_{i}-\frac{2R_{i}}{1+\exp\left(-p_{i}\left(\left|s_{i}\right|-\zeta_{i}\right)\right)}sig{\left(s_{i}\right)}^{m_{i}}-\frac{2K_{i}}{1+\exp\left(q_{i}\left(\left|s_{i}\right|-\zeta_{i}\right)\right)}sig{\left(s_{i}\right)}^{n_{i}}\;-Jsign\left(s_{i}\right)$$

To investigate the finite-time stability of the control system, the Lyapunov candidate is selected as

(35) $$V_{3_{i}}=0.5s_{i}^{2}$$

Then, taking the time derivative of $V_{3_{i}}$ and using Equation (34), we can obtain as

(36) $$\begin{array}{c} {\dot{V}}_{3_{i}}=s_{i}{\dot{s}}_{i}\\ =s_{i}\left(D_{i}-{\hat{D}}_{i}-\frac{2R_{i}}{1+\exp\left(-p_{i}\left(\left|s_{i}\right|-\zeta_{i}\right)\right)}sig{\left(s_{i}\right)}^{m_{i}}-\frac{2K_{i}}{1+\exp\left(q_{i}\left(\left|s_{i}\right|-\zeta_{i}\right)\right)}sig{\left(s_{i}\right)}^{n_{i}}-Jsign\left(s_{i}\right)\right)\\ \leq \left({\tilde{D}}_{i}-J\right)\left|s_{i}\right|-\frac{2R_{i}}{1+\exp\left(-p_{i}\left(\left|s_{i}\right|-\zeta_{i}\right)\right)}{\left|s_{i}\right|}^{m_{i}+1}-\frac{2K_{i}}{1+\exp\left(q_{i}\left(\left|s_{i}\right|-\zeta_{i}\right)\right)}{\left|s_{i}\right|}^{n+1}\\ \leq -\frac{2R_{i}}{1+\exp\left(-p_{i}\left(\left|s_{i}\right|-\zeta_{i}\right)\right)}{\left|s_{i}\right|}^{m_{i}+1}-\frac{2K_{i}}{1+\exp\left(q_{i}\left(\left|s_{i}\right|-\zeta_{i}\right)\right)}{\left|s_{i}\right|}^{n+1} \end{array}$$

We can see that, $V_{3_{i}}>0$ and ${\dot{V}}_{3_{i}}<0$ has been satisfied according to Lyapunov theory, so the control system is globally stable. To prove the control system that is globally finite-time stable, Equation (36) is rewritten as follows:

(37) $$s_{i}{\dot{s}}_{i}\;\leq -\frac{2R_{i}}{1+\exp\left(-p_{i}\left(\left|s_{i}\right|-\zeta_{i}\right)\right)}{\left|s_{i}\right|}^{m_{i}+1}-\frac{2K_{i}}{1+\exp\left(q_{i}\left(\left|s_{i}\right|-\zeta_{i}\right)\right)}{\left|s_{i}\right|}^{n+1}$$

From Equation (37), when $\left|s_{i}\left(0\right)\right|>\zeta_{i}$, the reaching phase is divided into two stages: $s_{i}\left(0\right)\to \left|s_{i}\right|=\zeta_{i}$ and $\left|s_{i}\right|=\zeta_{i}\to 0$. As a result, doing the same procedure with the sliding phase in Equations (24) and (25), the reaching time is calculated as:

(38) $$T_{r}=\underset{1\leq i\leq n}{max}\left\{t_{r1_{i}}+t_{r2_{i}}\;\right\}=\underset{1\leq i\leq n}{max}\left\{\frac{1}{R_{i}\left(-m_{i}+1\right)}\left({\left|s_{i}\left(0\right)\right|}^{-m_{i}+1}-\zeta_{i}{}^{-m_{i}+1}\right)+\frac{1}{K_{i}\left(-n_{i}+1\right)}\zeta_{i}{}^{-n_{i}+1}\right\}$$

Therefore, the control system is globally finite-time stable and the total convergence time for the system (11) is calculated by

(39) $$T_{c}=T_{o}+T_{r}+T_{s}$$

where $T_{o}$ is convergence time of FDO.

The proof is completed. □

## 4. Simulation Results and Discussion

**Figure 2:**
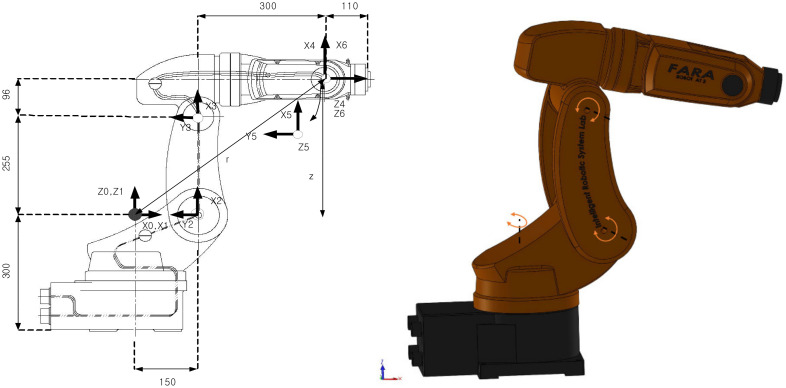
FARA ROBOT AT2 3-DOF robotic mechanical model in SOLIDWORK and its geometrical dimensions.

To verify the effectiveness of the proposed controller, we applied it to a FARA ROBOT AT2 3-DOF robotic manipulator [8]. To present the simulation results briefly, the robot system only simulated the first three links, the last three links of the robot were locked. We used MATLAB/SIMULINK software for all simulations. The configuration of the SIMULINK environment was set under a fixed-step (ODE5 dormand-prince) with 0.001 s system cycle time. All mechanical parts of the robotic manipulator were designed on SOLIDWORK software, then embedded into MATLAB/SIMULINK environment through the SIMSCAPE MULTIBODY LINK tool. Therefore, the simulation model of the robotic manipulator is the same as the actual mechanical model. External disturbances components and friction forces have been added to the robot system. Figure 2 shows the mechanical model of FARA ROBOT AT2 3-DOF in SOLIDWORK software and its geometrical dimensions, and Table 2 shows the design parameters of the robot system.

To verify the superiority of the proposed control system, its control performance is compared with other FTC methods including CTC-based FTC, SMC-based FTC, and FTSMC-based FTC in the aspects of convergence speed, position tracking control accuracy, and control inputs signals. To call shortened names of compared control methods, “CTC-based FTC” is replaced by “CTC”, “SMC-based FTC” is replaced by “SMC”, and “FTSMC-based FTC” is replaced by “FTSMC”.

The control input of CTC-based FTC is expressed as follows [36]:

(40) $$u=b^{-1}\left({\ddot{x}}_{d}-P\left(x\right)-K_{p}e-K_{d}\dot{e}\right)$$

where $K_{p}=diag\left\{K_{p_{1}},\;\ldots ,K_{p_{n}}\;\right\}$, $K_{p_{i}}>0$, $K_{d}=diag\left\{K_{d_{1}},\;\ldots ,K_{d_{n}}\;\right\}$, $K_{d_{i}}>0$.

The control input of SMC-based FTC is stated as follows [50]:

(41) $$\left\{\begin{array}{c} s=\dot{e}+ce\\ u=b^{-1}\left({\ddot{x}}_{d}-P\left(x\right)-c\dot{e}-\left(\Omega_{1}+\varsigma \right)sign(s)-\delta s\right) \end{array}\right.$$

where s is the linear sliding mode surface, $c=diag\left\{c_{1},\ldots ,\;c_{n}\right\}$ with $c_{i}$ is a positive constant, $\delta =diag\left\{\delta_{1},\ldots ,\;\delta_{n}\right\}$ with $\delta_{i}$ is a positive constant and ς is a small positive constant.

The control input of FTSMC-based FTC is described as follows [49]:

(42) $$\left\{\begin{array}{c} s=\dot{e}+\lambda sig{\left(e\right)}^{\alpha }+\omega sig{\left(e\right)}^{\beta }\\ u=b^{-1}\left({\ddot{x}}_{d}-P\left(x\right)-Z-\left(\Omega_{1}+\varsigma \right)sign(s)-\delta s\right)\; \end{array}\right.$$

where s is an FTSM surface, $\lambda =diag\left\{\lambda_{1},\;\ldots ,\;\lambda_{n}\right\},\;\lambda_{i}>0$, $\omega =diag\left\{\omega_{1},\;\ldots ,\;\omega_{n}\right\},\;\omega_{i}>0$, α and β are parameter vectors with the element as $\alpha_{i}>1$, $0<\beta_{i}<1$, $\delta =diag\left\{\delta_{1},\ldots ,\;\delta_{n}\right\}$ with $\delta_{i}$ is a positive constant, ς is a small positive constant, and $Z=\left[Z_{1},\;\ldots ,\;Z_{n}\right]$ with $Z_{i}=\alpha_{i}\lambda_{i}{\left|e_{i}\right|}^{\alpha_{i}-1}{\dot{e}}_{i}+\beta_{i}\omega_{i}{\left|e_{i}\right|}^{\beta_{i}-1}{\dot{e}}_{i}$.

The robot’s end-effector is controlled to follow the desired trajectory, as described below

(43) $$\left[\begin{array}{c} x_{d}\\ y_{d}\\ z_{d} \end{array}\right]=\left[\begin{array}{c} 0.43+0.01\sin\left(\frac{t}{4}\right)\\ 0.006\sin\left(\frac{t}{4}\right)\\ 0.26+0.006\cos\left(\frac{t}{8}\right) \end{array}\right]\left(m\right)$$

The friction forces are modeled by

(44) $$F\left(\dot{\theta }\right)=\left[\begin{array}{c} 2{\dot{\theta }}_{1}+0.01sign({\dot{\theta }}_{1})\\ 2{\dot{\theta }}_{2}+0.01sign({\dot{\theta }}_{2})\\ 2{\dot{\theta }}_{3}+0.01sign({\dot{\theta }}_{3}) \end{array}\right]$$

The external disturbances are added to the system as follows:

(45) $$\tau_{d}=\left[\begin{array}{c} -5\left(1-\exp\left(-0.4t\right)\right)-0.3\sin(0.8t)\\ -3\left(1-\exp\left(-0.4t\right)\right)+0.1\sin(0.5t)\\ -1.8\left(1-\exp\left(-0.4t\right)\right)-0.1\sin(1.6t) \end{array}\right]$$

The uncertainty components of the dynamic model are assumed as:

(46) $$\left\{\begin{array}{c} \Delta M=0.15M\\ \Delta C=0.15C\\ \Delta G=0.15G \end{array}\right.$$

The root-mean-square errors (RMSEs) are given as:

(47) $$E_{X}=\sqrt{\frac{1}{K}\overset{K}{\underset{i=1}{\sum }}{\left(x_{d_{i}}-x_{i}\right)}^{2}},\;E_{Y}=\sqrt{\frac{1}{K}\overset{K}{\underset{i=1}{\sum }}{\left(y_{d_{i}}-y_{i}\right)}^{2}},\;E_{Z}=\sqrt{\frac{1}{K}\overset{K}{\underset{i=1}{\sum }}{\left(z_{d_{i}}-z_{i}\right)}^{2},}\;E_{1}=\sqrt{\frac{1}{K}\overset{K}{\underset{i=1}{\sum }}{\left(\theta_{d1_{i}}-\theta_{1_{i}}\right)}^{2}},\;E_{2}=\sqrt{\frac{1}{K}\overset{K}{\underset{i=1}{\sum }}{\left(\theta_{d2_{i}}-\theta_{2_{i}}\right)}^{2}},\;E_{3}=\sqrt{\frac{1}{K}\overset{K}{\underset{i=1}{\sum }}{\left(\theta_{d3_{i}}-\theta_{3_{i}}\right)}^{2}}$$

where K is the number of samples to be considered in this calculation. $x_{d}{}_{i},\;y_{d_{i}},\;z_{d}{}_{i}$ and $x_{i},\;y_{i},\;z_{i}$ represent the desired trajectory and the real trajectory of end-effector in XYZ-directions at the time index i, respectively. $\theta_{d1}{}_{i},\;\theta_{d2}{}_{i},\;\theta_{d3}{}_{i}$ and $\theta_{1_{i}},\;\theta_{2_{i}},\;\theta_{3_{i}}$ stand for the desired joint angle and the real joint angle of three-joints at the time index i, respectively.

The selected parameters of the controllers are shown in Table 3.

To facilitate the evaluation of tracking errors, RMSEs are calculated according to Equation (47) over a period of 2nd to 40th s. The results are presented in Table 4 and Table 5.

To prove the validity of the proposed system, simulations have been performed in the two cases below.

**Case 1:** This simulation considers the robot in normal operating conditions. Therefore, the robot system is only affected by the uncertainty components such as friction force, uncertainties of the dynamic model, and external disturbance. There is no fault occurrence in this case.

**Figure 3:**
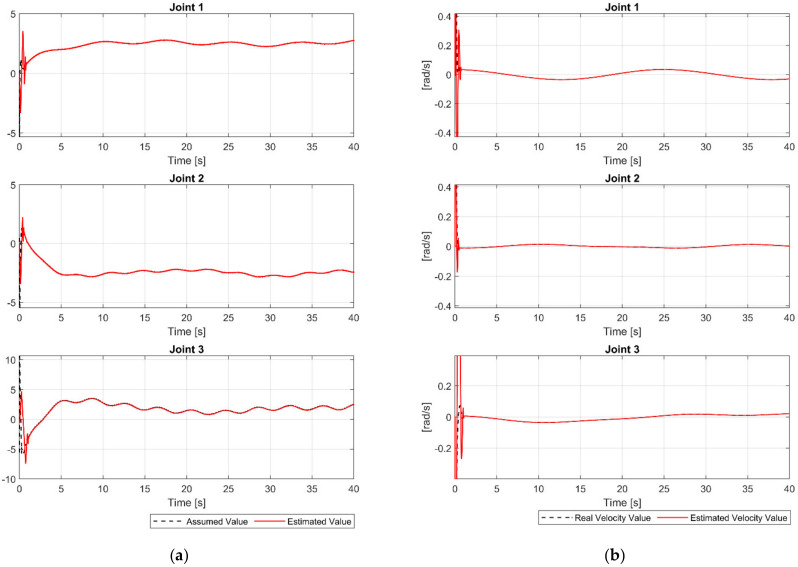
(**a**) The assumed value of uncertainty and its estimated value at joints; (**b**) the real velocity value and its estimated value at joints.

The main target of the proposed FDO is used to detect and estimate the whole uncertainty components. The time history of assumed uncertainty components and the outputs of the proposed FDO are shown in Figure 3a. We can clearly see in Figure 3a that, the proposed FDO provided a high precision estimation of the uncertainty components. In addition, we can see from Figure 3b that the proposed FDO also provided a highly accurate estimate of the velocities of the joints. From the accurately estimated information of the proposed observer, the performance of the control system is greatly improved in increasing tracking accuracy and reducing chattering.

**Figure 4:**
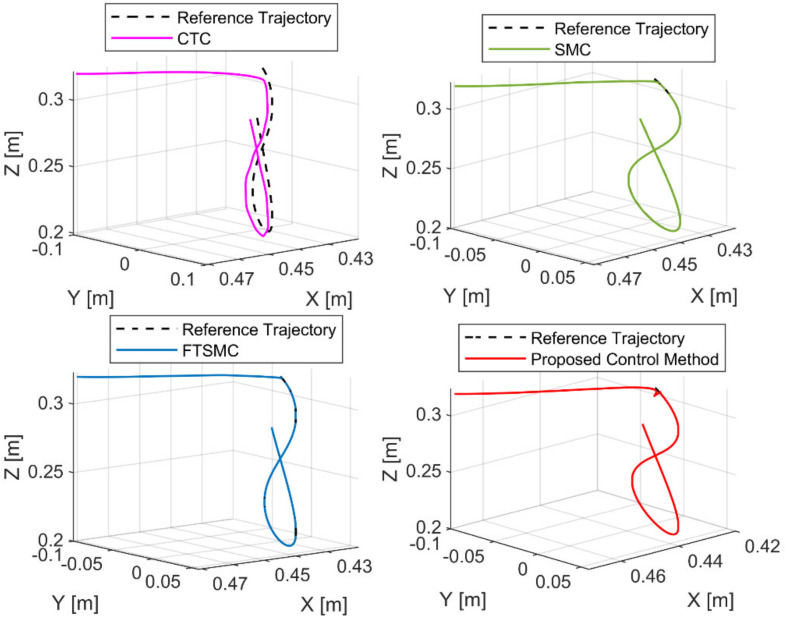
The reference trajectory and real trajectory of the end-effector under four controllers.

**Figure 5:**
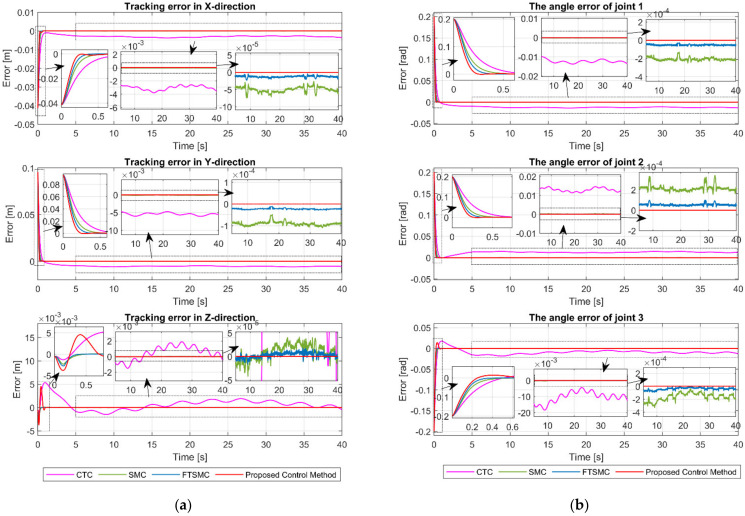
(**a**) The tracking errors of end-effector in X, Y, and Z direction; (**b**) the tracking errors of joint 1, joint 2, and joint 3.

Figure 4 shows the path tracking performances of the end-effector under four controllers. From Figure 4, we can see that CTC provided a poor tracking performance when the system was heavily influenced by uncertain components. Due to the robustness to the uncertainty components of SMC and FTSMC, we can see that SMC and FTSMC provided a good tracking performance. From the correct information of the proposed FDO, the proposed controller provided a good position tracking performance, as shown in Figure 4. For a more detailed analysis of the tracking performance, the tracking errors were presented in Figure 5 and Table 4.

Figure 5a,b, respectively, show the tracking error of the end-effector in XYZ-space and the angle tracking error at the joints. From Figure 5 and the results in Table 4, we can clearly see that CTC provided the worst tracking error among the four controllers. CTC’s position tracking errors in XYZ-space are 0.0030, 0.0054, and 0.0011, and its angle tracking errors at joints are 0.0121, 0.0127, and 0.0106, respectively. As we can expect, the SMC provided a smaller tracking error than the CTC due to its robustness to uncertainty components. XYZ-space position tracking errors of SMC are $4.9770\times 10^{-5}$, $8.9695\times 10^{-5}$, and $1.9887\times 10^{-5}$, whereas its joint angle tracking errors are $2.0411\times 10^{-4}$, $2.1125\times 10^{-4}$, and $1.7078\times 10^{-4}$, respectively. Nevertheless, the tracking error of SMC is worse than that of FTSMC. As shown in Table 4, the FTMC has position tracking errors of $1.2499\times 10^{-5}$, $2.2562\times 10^{-5}$, and $5.9538\times 10^{-6}$ in XYZ-space and angle tracking errors of $5.1323\times 10^{-5}$, $5.3539\times 10^{-5}$, and $4.2223\times 10^{-5}$ at joints. We can easily see that the proposed controller offered a superior tracking performance among the four controllers. The proposed controller’s position tracking errors in XYZ-space are $1.4083\times 10^{-8}$, $2.6478\times 10^{-8}$, and $2.3604\times 10^{-8}$, and its angle tracking errors at joints are $6.1299\times 10^{-8}$, $5.5757\times 10^{-8}$, and $5.5795\times 10^{-8}$, respectively. Furthermore, we can see in Figure 5 that the proposed controller provided the fastest convergence rate.

**Figure 6:**
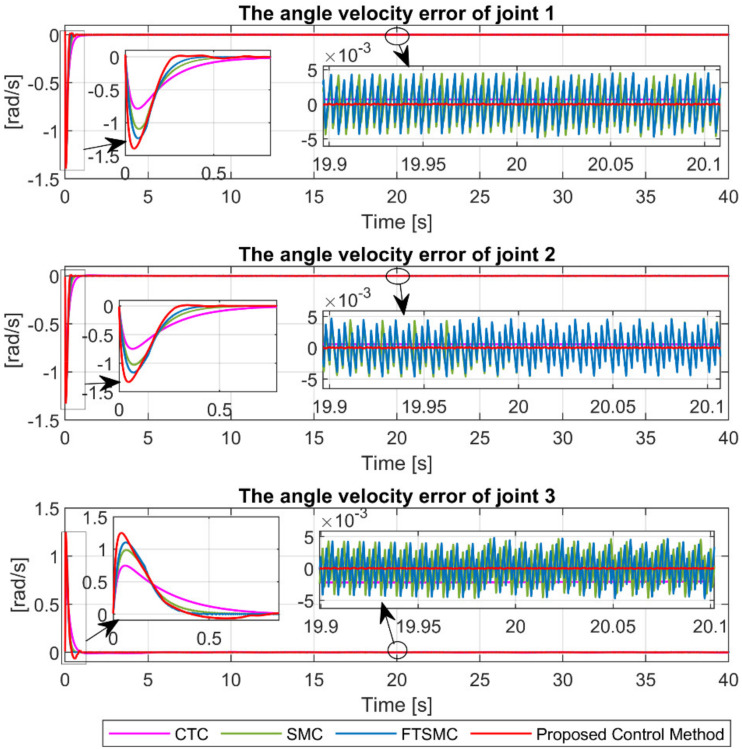
The tracking velocity errors of joint 1, joint 2, and joint 3.

The angular velocity error at the joints under the four controllers is shown in Figure 6. It is remarkable that the proposed control method provides the smallest velocity control error and the fastest convergence speed compared to the remaining controllers.

**Figure 7:**
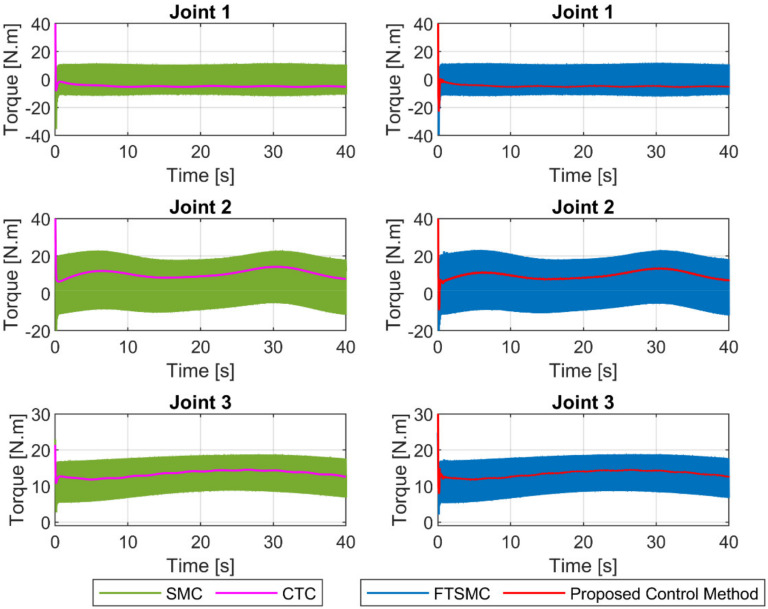
The control input torques at joints of four controllers.

The control torques at the joints generated by the four controllers were illustrated in Figure 7. We can clearly see that the SMC and FTSMC provided discontinuous control signals since both controllers were applied a large sliding gain ($\Omega_{1}+\varsigma$) in the reaching control term ($\left(\Omega_{1}+\varsigma \right)sign(s)$) to counteract the influences of uncertainty components. The CTC provided a continuous control signal since there is no $sign\left(\cdot \right)$ function in its control input. Particularly, the proposed controller provided a smooth control signal, as illustrated in Figure 7. Since the entire uncertainty component was estimated by the proposed observer. Therefore, only a small value of the sliding gain in the reaching control term ($u_{r}$) was used to compensate for the observer’s estimation error.

**Case 2:** The robot system is affected not only by the uncertainty components as in case 1 but also by faults.

A fault function is supposed to illustrate the effect of the fault in the robot system as follows:

(48) $$\Gamma \left(\theta ,\dot{\theta },\tau \right)=\left[\begin{array}{cc} \left(1-\exp\left(-\left(t-15\right)\right)\right)\left(5-0.5\sin\left(2\left(t-15\right)\right)\tau_{1}\right) & t\geq 15\\ \left(1-\exp\left(-\left(t-20\right)\right)\right)\left(3-0.4\sin\left(1.5\left(t-20\right)\right)\tau_{2}\right) & t\geq 20\\ \left(1-\exp\left(-\left(t-25\right)\right)\right)\left(2-0.1\sin\left(2.2\left(t-25\right)\right)\tau_{3}\right) & t\geq 25 \end{array}\right]$$

Equation (48) shows that a fault $\left(1-\exp\left(-\left(t-15\right)\right)\right)\left(5-0.5\sin\left(2\left(t-15\right)\right)\tau_{1}\right)$ will occur in the first joint at time t≥ 15 s, other faults $\left(1-\exp\left(-\left(t-20\right)\right)\right)\left(3-0.4\sin\left(1.5\left(t-20\right)\right)\tau_{2}\right)$, $\left(1-\exp\left(-\left(t-25\right)\right)\right)\left(2-0.1\sin\left(2.2\left(t-25\right)\right)\tau_{3}\right)$ which are assumed to respectively occur in the second joint and third joint at the times t≥ 20 s and t≥ 25 s.

**Figure 8:**
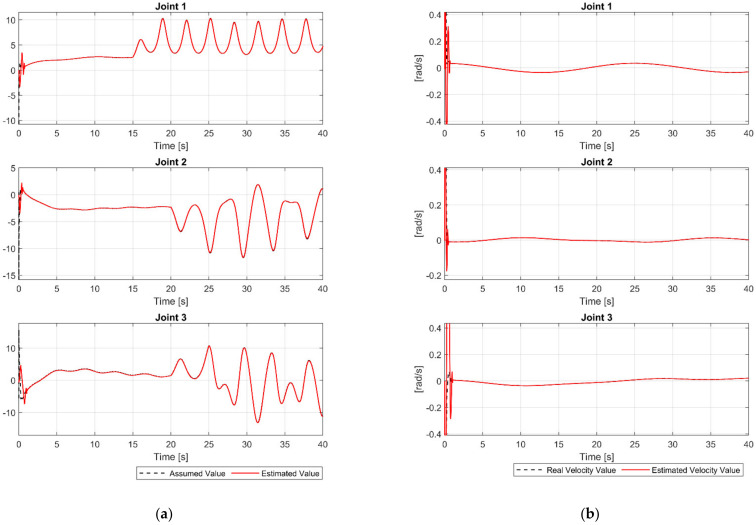
(**a**) The assumed value of uncertainty and fault, and its estimated value at joints; (**b**) the real velocity value and its estimated value at joints.

From Figure 8a, we can see that the proposed observer also provided a very high accuracy estimation of uncertainty and fault components as in case 1. By utilizing the proposed FDO’s accurate fault information, the control system performance was significantly improved when the faults occur in the robot system. Furthermore, the velocity values at the joints were also accurately estimated as illustrated in Figure 8b.

**Figure 9:**
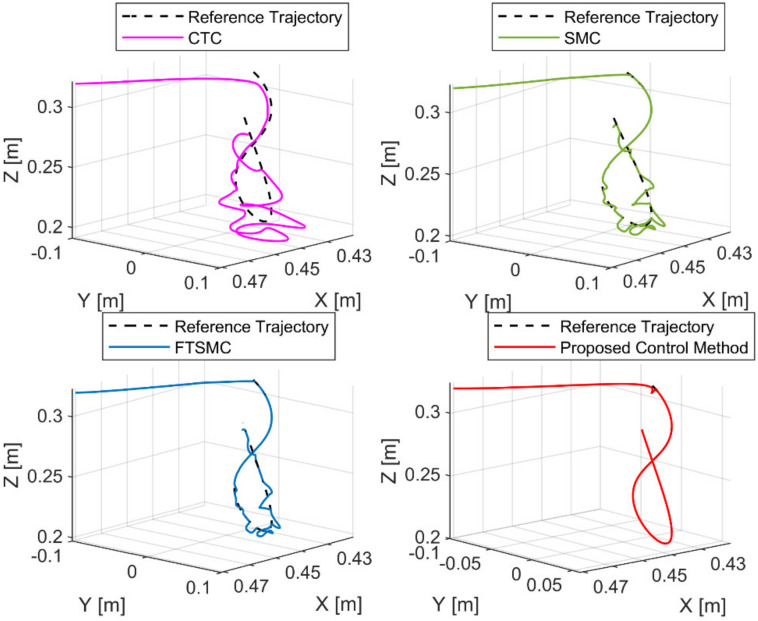
The reference trajectory and real trajectory of the end-effector under four controllers.

**Figure 10:**
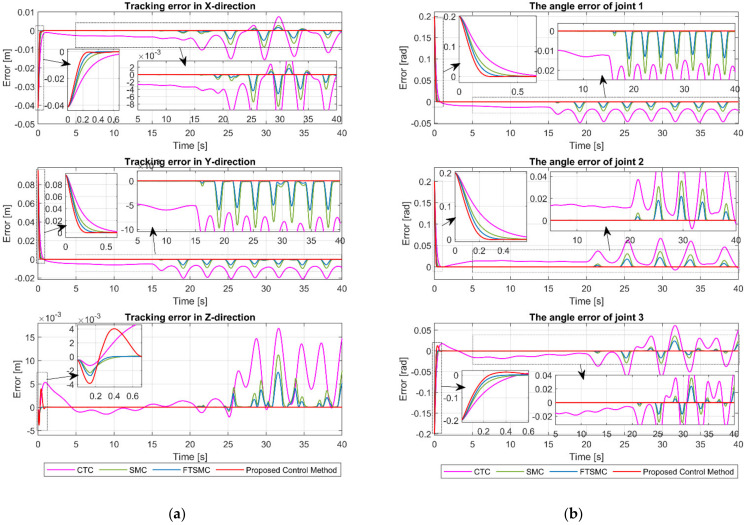
(**a**) The tracking errors of end-effector in X, Y, and Z direction.; (**b**) The tracking errors of joint 1, joint 2, and joint 3.

The tracking performance in the cartesian space and the tracking errors are respectively exhibited in Figure 9 and Figure 10. As shown in Figure 9 and Figure 10, the CTC exhibits its powerlessness against the effect of fault, and it was unable to maintain desired tracking performance when faults occur. As reported in Table 5, CTC’s RMSEs in XYZ-space are 0.0056, 0.0108, and 0.0055, and its RMSEs at joints are 0.0247, 0.0234, and 0.0234, respectively. It can be easily seen that the SMC provided better fault tolerance and transient response than the CTC, whereas the FTSMC offered better fault tolerance and transient response than the SMC. However, SMC and FTMSC do not provide a good tracking performance when faults appear seriously, as shown in Figure 10. The results in Table 5 clearly demonstrate that the tracking performance of SMC and FTSMC decreases significantly when a sufficiently large fault occurs. Specifically, SMC’s RMSEs in XYZ-space are 0.0019, 0.0031, and 0.0020, and its RMSEs at joints are 0.0072, 0.0078, and 0.0076, respectively, whereas FTSMC’s RMSEs in XYZ-space are 0.0011, 0.0018, and 0.0013, and its RMSEs at joints are 0.0042, 0.0045, and 0.0045, respectively. By using precisely estimated fault information from the proposed FDO, the proposed control algorithm provided superior fault tolerance and transient response compared to the three remaining controllers. We can easily see from Figure 9 and Figure 10 and Table 5 that the proposed controller was able to maintain good tracking performance despite the occurrence of the fault. The RMSEs of the proposed controller in XYZ-space are $4.4172\times 10^{-8}$, $7.7403\times 10^{-8}$, and $6.7581\times 10^{-8}$, and its RMSEs at joints are $1.7940\times 10^{-7}$, $1.6488\times 10^{-7}$, and $2.3066\times 10^{-7}$, respectively.

**Figure 11:**
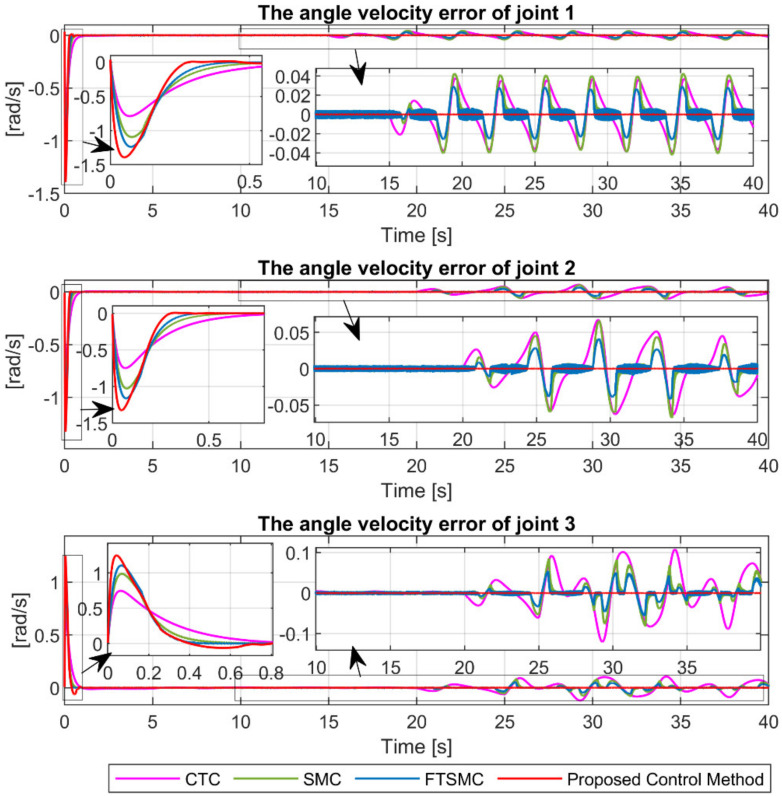
The tracking velocity errors of joint 1, joint 2, and joint 3.

Figure 11 illustrates the angular velocity error at the joints under the four controllers. We can easily see that the proposed controller also provides superior velocity control accuracy and the fastest convergence among the four controllers in case of fault occurrence.

**Figure 12:**
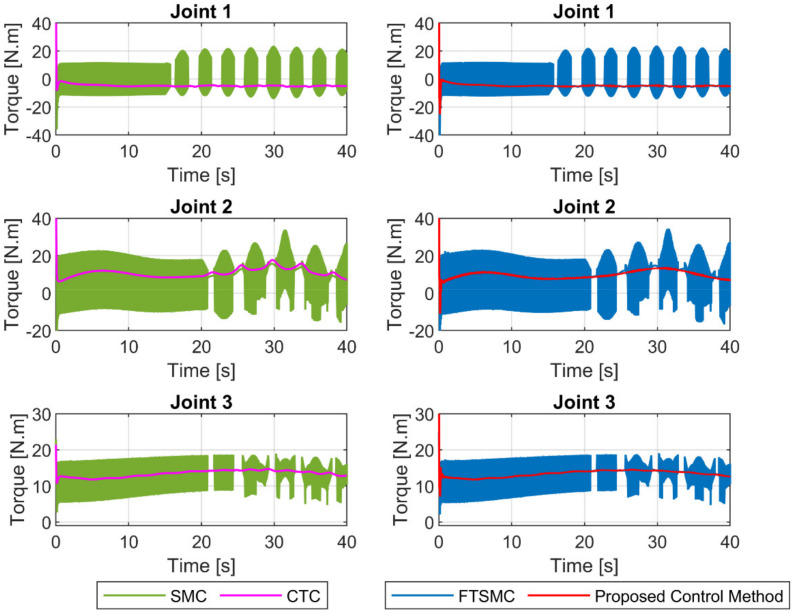
The control input torques at joints under four controllers.

A summary of the control input torques at the joints of four controllers is illustrated in Figure 12. It is seen that the CTC provided a continuous control signal, whereas the SMC and the FTSMC provided a discontinuous control signal, as discussed in case 1. It is noteworthy that the proposed control method provided a smooth control signal in both cases.

From the results presented in the two cases above, we can conclude that the proposed control strategy provided outstanding performance in terms of tracking error accuracy, fast convergence speed, smooth control torque, and strong fault-tolerant ability compared to the three remaining controllers.

**Remark** **2:** *From the theoretical analysis and comparative simulation results, we can see that the advantages of the proposed FTC over conventional FD/FTC such as CTC-based FTC, SMC-based FTC, and FTSMC-based FTC in the aspects of convergence speed, position tracking control accuracy, and control input signals are given as:*

- *The proposed control system provides a faster convergence rate for both observation error and control error and guarantees convergence in finite time*.
- *The proposed control method provides higher control precision and stronger against disturbance, uncertainties, and faults. Thus, it can maintain the desired performance for the system in case of faults*.
- *The proposed controller provides smoother control signals compared to conventional SMC and conventional FTSMC with a significant reduction of chattering behavior. This minimizes friction between moving mechanical components and reduces heat generation in the power circuit. As a result, it prolongs the life of devices*.

## 5. Conclusions

In this paper, an FTC system has been developed based on the combination of a finite time observer and advanced TSMC for robot manipulators. The proposed FTC system provided a fast convergence rate for both observation error and control error in finite time. The stability and finite-time convergence of the proposed control system have been verified such that they have been always strictly guaranteed by Lyapunov theory and finite-time control theory.

Through the obtained results from the theory of control design and the comprehensive comparisons with some different FTC methods for a FARA robotic manipulator, the proposed FTC has been confirmed that it is capable of detecting, approximating, and eliminating the influence of some faults occurring in the robot. The operation of the robot system has been always guaranteed with expected performance and maintained that performance even in case of faults, including high tracking accuracy, small chattering behavior, and fast transient response with a variation of disturbances, uncertainties, or faults.

In this paper, data such as angular position or angular velocity from the measuring sensors are assumed to be unaffected by measurement noise or faults when the proposed FTC method was designed. The mentioned problems will be fully considered in our subsequent studies.

## Figures and Tables

**Table 1:** List of symbols.

Notation	Description
${\mathbb{R}}^{n}$	n-dimensional real vector space
${\mathbb{R}}^{n\times n}$	n×n matrix whose entries are real numbers
$\left|\cdot \right|$	absolute value of
θ	vector of joint angular position, $\theta \in {\mathbb{R}}^{n}$
$\dot{\theta }$	vector of joint angular velocity, $\dot{\theta }\in {\mathbb{R}}^{n}$
$\ddot{\theta }$	vector of joint angular acceleration, $\ddot{\theta }\in {\mathbb{R}}^{n}$
$M\left(\theta \right)$	matrix of actual inertia, $M\left(\theta \right)=\hat{M}\left(\theta \right)+\Delta M\left(\theta \right)\in {\mathbb{R}}^{n\times n}$
$C\left(\theta ,\dot{\theta }\right)$	matrix of the actual centrifugal and Coriolis force, $C\left(\theta ,\dot{\theta }\right)=\hat{C}\left(\theta ,\dot{\theta }\right)+\Delta C\left(\theta ,\dot{\theta }\right)\in {\mathbb{R}}^{n\times n}$
$G\left(\theta \right)$	vector of actual gravity, $G\left(\theta \right)=\hat{G}\left(\theta \right)+\Delta G\left(\theta \right)\in {\mathbb{R}}^{n}$
$\hat{M}\left(\theta \right)$	estimated matrix of $M\left(\theta \right)$, $\hat{M}\left(\theta \right)\in {\mathbb{R}}^{n\times n}$
$\hat{C}\left(\theta ,\dot{\theta }\right)$	estimated matrix of $C\left(\theta ,\dot{\theta }\right)$, $\hat{C}\left(\theta ,\dot{\theta }\right)\in {\mathbb{R}}^{n\times n}$
$\hat{G}\left(\theta \right)$	estimated matrix of $G\left(\theta \right)$, $\hat{G}\left(\theta \right)\in {\mathbb{R}}^{n}$
$\Delta M\left(\theta \right)$	estimation error matrix of $M\left(\theta \right)$, $\Delta M\left(\theta \right)\in {\mathbb{R}}^{n\times n}$
$\Delta C\left(\theta ,\dot{\theta }\right)$	estimation error matrix of $C\left(\theta ,\dot{\theta }\right)$, $\Delta C\left(\theta ,\dot{\theta }\right)\in {\mathbb{R}}^{n\times n}$
$\Delta G\left(\theta \right)$	estimation error vector of $G\left(\theta \right)$, $\Delta G\left(\theta \right)\in {\mathbb{R}}^{n}$
$F\left(\dot{\theta }\right)$	vector of the friction force, $F\left(\dot{\theta }\right)\in {\mathbb{R}}^{n}$
$T_{d}\left(t\right)$	vector of external disturbance, $T_{d}\left(t\right)\in {\mathbb{R}}^{n}$
τ	vector of the control input torque, $\tau \in {\mathbb{R}}^{n}$
$\sigma \left(t-T_{F}\right)$	matrix of fault time profile, $\sigma \left(t-T_{F}\right)\in {\mathbb{R}}^{n\times n}$
$T_{F}$	vector of the time when the faults occur, $T_{F}\in {\mathbb{R}}^{n}$
$\ell_{i}$	the developing rate coefficient of the $i^{th}$ fault
$\Gamma \left(\theta ,\dot{\theta },\tau \right)$	matrix of the unexpected fault, $\Gamma \left(\theta ,\dot{\theta },\tau \right)\in {\mathbb{R}}^{n}$
$P\left(x\right)$	lumped nominal part of the robot, $P\left(x\right)\in {\mathbb{R}}^{n}$
$B\left(x\right)$	a smooth function, $B\left(x\right)\in {\mathbb{R}}^{n\times n}$
D	the whole uncertainties, external disturbances, and faults, $D\in {\mathbb{R}}^{n}$
$\Omega_{1}$, $\Omega_{2}$	the bounded values of D and $\dot{D}$**,** $\Omega_{1}$ and $\Omega_{2}$ are positive constants
${\hat{x}}_{1}$	the estimated value of the position $x_{1}$
${\hat{x}}_{2}$	the estimated value of the velocity $x_{2}$
$\hat{D}$	the estimated value of the whole uncertainty and fault D
${\tilde{x}}_{1}$	estimation error of position $x_{1}$
$\;{\tilde{x}}_{2}\;$	estimation error of velocity $x_{2}$
$\tilde{D}$	estimation error of the whole uncertainty and fault D
φ, ψ, ϱ	positive matrices
J	bounded valued of the estimation error of the whole uncertainty and fault $\tilde{D}$, J>0
$\theta_{d_{i}},\;{\dot{\theta }}_{d_{i}}$	desired position and desired velocity at $i^{th}$ joint
$x_{1_{i}}$, $x_{2_{i}}$	the actual position and actual velocity at $i^{th}$ joint
$x_{e_{i}},\;x_{de_{i}}$	tracking position error and the tracking velocity error at $i^{th}$ joint
$s_{i}$	sliding mode surface of $i^{th}$ joint
$\lambda_{i},\;\eta_{i},\;\omega_{i},\;\sigma_{i},\;$ $\alpha_{i},\;\beta_{i},\gamma_{i}$	parameters of sliding mode surface of $i^{th}$ joint, $\lambda_{i}>0,\eta_{i}>0,\omega_{i}>0,\sigma_{i}>0$, $\alpha_{i}>1$, $0<\beta_{i}<1$, $\gamma_{i}={\left(\omega_{i}/\lambda_{i}\right)}^{1/\left(\alpha_{i}-\beta_{i}\right)}$
$R_{i},\;p_{i},K_{i},\;q_{i},\;$ $m_{i},\;n_{i},\;\zeta_{i}\;$	parameters of reaching control law of $i^{th}$ joint, $R_{i}>0,\;p_{i}>0,\;K_{i}>0$, $q_{i}>0$ $m_{i}>1$, $0<n_{i}<1$, $\zeta_{i}={\left(K_{i}/R_{i}\right)}^{1/\left(m_{i}-n_{i}\right)}$
$K_{p},\;K_{d}$	parameters of CTC-based FTC, $K_{p}$ and $K_{d}$ are positive diagonal matrices
c, δ, ς	parameters of SMC-based FTC, c and δ are positive diagonal matrices, ς>0
$\lambda_{i},\;\omega_{i},\;\alpha_{i},\;\beta_{i}$, ς	parameters of FTSMC-based FTC, $\lambda_{i}>0,\;\omega_{i}>0$, $\alpha_{i}>1,\;0<\beta_{i}<1,$ ς>0
$x_{d},\;y_{d},\;z_{d}$	the desired trajectory of the robot’s end-effector in XYZ-space
$E_{X},\;E_{Y},\;E_{Z}$	the root-mean-square errors of the robot’s end-effector in XYZ-space
$E_{1},\;E_{2},\;E_{3}$	the root-mean-square errors of the robot’s joints

**Table 2:** The designed parameters of the FARA robot system.

		Link 1	Link 2	Link 3
Length (m)		0.15	0.255	0.3
Weight (kg)		37.985	21.876	16.965
Center of Mass (mm)	$l_{cx}$	68.067	95.045	71.496
	$l_{cy}$	−1.185	5.399	−72.007
	$l_{cz}$	64.931	−0.002	−1.004
Inertia (kg·m^2^)	$I_{xx}$	0.252	0.359	0.306
	$I_{yy}$	0.395	0.623	0.853
	$I_{zz}$	0.356	0.319	0.306

**Table 3:** The selected parameters of three controllers.

Controller	Symbol	Value
CTC	$K_{p},\;K_{d}$	$diag\left\{200,200,200\right\},\;diag\left\{40,40,40\right\}$
SMC	$c,\delta ,\;\Omega_{1},\;\varsigma$	$diag\left\{10,10,10\right\},\;diag\left\{20,20,20\right\},\;5.5,\;0.01$
FTSMC	$\lambda_{i},\omega_{i},\alpha_{i},\;\beta_{i},\delta ,\;\Omega_{1},\varsigma$	$5,\;1.01,\;5,\;0.8,\;diag\left\{20,20,20\right\},\;5.5,\;0.01$
Proposed Method	$\lambda_{i},\eta_{i},\omega_{i},\sigma_{i}$, $\alpha_{i}$, $\beta_{i}$ $\varphi_{i},\;\psi_{i},\varrho_{i}$ $R_{i},\;p_{i}$, $K_{i}$$,\;q_{i},\;m_{i}$$,\;n_{i}$, J	5, 1.1, 5, 2.2, 1.01, 0.8 5.604, 16.645, 24.2 10, 1.1, 10, 1.1, 1.8, 0.7, 0.1

Note: i=1, 2, 3.

**Table 4:** RMSEs of four controllers in the first case.

Controller	$E_{x}$ (X-Direction)	$E_{y}$ (Y-Direction)	$E_{z}$ (Z-Direction)	$E_{1}$ (Joint 1)	$E_{2}$ (Joint 2)	$E_{3}$ (Joint 3)
CTC	0.0030	0.0054	0.0011	0.0121	0.0127	0.0106
SMC	$4.9770\times 10^{-5}$	$8.9695\times 10^{-5}$	$1.9887\times 10^{-5}$	$2.0411\times 10^{-4}$	$2.1125\times 10^{-4}$	$1.7078\times 10^{-4}$
FTSMC	$1.2499\times 10^{-5}$	$2.2562\times 10^{-5}$	$5.9538\times 10^{-6}$	$5.1323\times 10^{-5}$	$5.3539\times 10^{-5}$	$4.2223\times 10^{-5}$
Proposed Method	$1.4083\times 10^{-8}$	$2.6478\times 10^{-8}$	$2.3604\times 10^{-8}$	$6.1299\times 10^{-8}$	$5.5757\times 10^{-8}$	$5.5795\times 10^{-8}$

**Table 5:** RMSEs of four controllers in the second case.

Controller	$E_{x}$ (X-Direction)	$E_{y}$ (Y-Direction)	$E_{z}$ (Z-Direction)	$E_{1}$ (Joint 1)	$E_{2}$ (Joint 2)	$E_{3}$ (Joint 3)
CTC	0.0056	0.0108	0.0055	0.0247	0.0234	0.0234
SMC	0.0019	0.0031	0.0020	0.0072	0.0078	0.0076
FTSMC	0.0011	0.0018	0.0013	0.0042	0.0045	0.0045
Proposed Method	$4.4172\times 10^{-8}$	$7.7403\times 10^{-8}$	$6.7581\times 10^{-8}$	$1.7940\times 10^{-7}$	$1.6488\times 10^{-7}$	$2.3066\times 10^{-7}$

## Data Availability

The data sets generated and/or analyzed during the current study are available from the corresponding author on reasonable request.
